# Complex Regional Pain Syndrome After Herpes Zoster Infection: An Uncommon and Underrecognized Clinical Entity

**DOI:** 10.7759/cureus.108874

**Published:** 2026-05-15

**Authors:** Anurag Mantri, Venkata Kaumudi Ayaluri, Harshitha Reddy, Nissy Jose

**Affiliations:** 1 Department of Public Health, York St John University, London, GBR; 2 Department of Accident and Emergency, Bronglais General Hospital, Aberystwyth, GBR; 3 Department of Internal Medicine, Navjeevan Hospital, Hyderabad, IND; 4 Department of Emergency Medicine, Bronglais General Hospital, Aberystwyth, GBR

**Keywords:** budapest criteria, complex regional pain syndrome, herpes zoster, neuropathic complications, post-herpetic oedema, varicella-zoster

## Abstract

Latent varicella-zoster virus reactivation causes herpes zoster, which manifests as a dermatomal rash. Complex regional pain syndrome (CRPS) after herpes is an uncommon complication in clinical practice. CRPS has the following main components, which include pain, sensory, vasomotor, sudomotor, and motor/trophic. The most common sequelae of herpes zoster are postherpetic neuralgia, but CRPS is underrecognised.

We present a case report of a 68-year-old female patient who had vesicular rash along the C7/C8 dermatome, clinically indicating a herpes zoster episode. Following this episode, she experienced pain, swelling, and functional impairment in the C7, C8 dermatome. CRPS was diagnosed on the basis of the Budapest criteria clinically. Neuropathic pain drugs, physical therapy, and supportive care are all part of a multidisciplinary strategy that gradually improves symptoms. This case highlights a rare herpes zoster infection sequelae of CRPS that remains underrecognised in clinical practice in developing countries. Post-herpetic neuralgia is a common sequela, and its progression to CRPS represents a distinct and underdiagnosed cause in patients.

## Introduction

Complex regional pain syndrome (CRPS) is characterised by severe, disproportionate regional pain accompanied by sensory, vasomotor, sudomotor, and motor disturbances. It is typically classified into Type I (no confirmed nerve injury) and Type II (nerve lesion is present) [1]. The condition most commonly follows trauma or surgery; however, non-traumatic triggers such as infections have also been implicated [2].

Herpes zoster is a painful vesicular eruption with a dermatomal distribution that is brought on by the reactivation of latent varicella-zoster virus (VZV) in the dorsal root ganglia [3]. It is frequently associated with complications such as postherpetic neuralgia (PHN), particularly in older or immunocompromised individuals. PHN is characterised by persistent neuropathic pain following resolution of the rash and represents the most common long-term sequela of herpes [4].

In contrast, the development of CRPS following herpes zoster is rare and not well recognised. There is an overlap of symptoms between CRPS and PHN. However, CRPS is distinguished by additional clinical features such as oedema, changes in skin colour and temperature, sudomotor dysfunction, and motor impairment, which reflect the underlying autonomic and inflammatory dysregulation apart from pain, which is most common in PNH [5]. The pathophysiological mechanisms are likely to include a combination of peripheral nerve injury, neuroinflammation, central sensitisation, and immune-mediated processes. Viral-induced damage to sensory neurons may initiate a cascade of events, leading to abnormal pain processing and autonomic dysfunction, ultimately resulting in CRPS [6]. Differential diagnosis includes cellulitis, Inflammatory arthritis, and postherpetic neuralgia. Cellulitis was considered less likely due to the absence of progressive erythema, purulent lesions, systemic toxicity, fever, or leukocytosis. Inflammatory arthritis was unlikely given the absence of joint-specific swelling, deformity, and prolonged morning stiffness. 

## Case presentation

A 68-year-old female patient, a known hypertensive on regular medication, presented with complaints of swelling and stiffness of the right hand for two weeks. She experienced a severe vesicular rash involving the right C7, C8 dermatome four weeks before this presentation. The rash was associated with burning pain and was clinically diagnosed as herpes zoster. The skin lesions disappeared over the course of 10-12 days after she was given oral acyclovir (800 mg five times daily) for seven days. However, she had continuous pain even after the resolution of the rash. With a Visual Analog Scale (VAS) score of 8/10, the pain was characterised as acute, searing, and constant over the course of the following two weeks. The pain was associated with marked hypersensitivity to touch (allodynia) and exaggerated response to painful stimuli (hyperalgesia). She also noticed a progressive swelling of the right hand, along with stiffness and difficulty in performing activities of daily living. There was no prior history of trauma or surgery. No history suggestive of inflammatory arthritis or connective tissue disease was noted. 

On examination, the right hand and distal forearm showed diffuse non-pitting oedema with mild erythema. Healed hyperpigmented lesions consistent with prior herpes zoster were noted along the right C7, C8 dermatomal distribution. Sensory examination revealed allodynia and hyperalgesia. However, a motor examination revealed that the wrist and finger joints had a limited range of motion because of pain. Grip strength was reduced. No focal neurological deficits were identified, and distal pulses are normal. Patient laboratory investigations, such as complete blood picture, kidney function, and liver function tests are shown in Table 1.

**Table 1:** Results of laboratory investigations

Laboratory investigations	Value	Biological reference range
Hemoglobin (g/dl)	10.3	13-15
Total leucocyte count (/cumm)	11100	4000-11,000
Platelet (/cumm)	5.23	1,50,000-4,50,000
Mean Corpuscular Volume (fl)	92	79-100
Urea (mg/dl)	18	9-20
Creatinine (mg/dl)	0.6	0.6-1.2
Sodium (mmol/l)	139	137-145
Potassium (mmol/l)	3.7	3.5-5.1
Alkaline phosphatase (units/l)	126	38-126
ALT (alanine aminotransferase) (u/l)	26	<50
AST (aspartate aminotransferase) (u/l)	39	17-59
Total protein (g/dl)	6.5	6.3-8.2
Albumin (g/dl)	3.7	3.5-5
Total bilirubin (mg/dl)	1.1	0.2-1.3
Conjugated bilirubin (mg/dl)	0.3	0-0.3
Unconjugated bilirubin (mg/dl)	0.8	0-1.1
Globulin (g/dl)	2.8	2.3-3.5

Budapest diagnostic criteria were used in the patient's diagnosis (Table 2).

**Table 2:** Budapest criteria applied in the patient

Budapest category	Findings in the patient
Sensory	Allodynia, Hyperalgesia
Vasomotor	Local warmth and erythema of the affected hand
Sudomotor	Swelling and oedema of the involved fingers and hand
Motor	Restricted finger movements due to pain and stiffness

**Figure 1:**
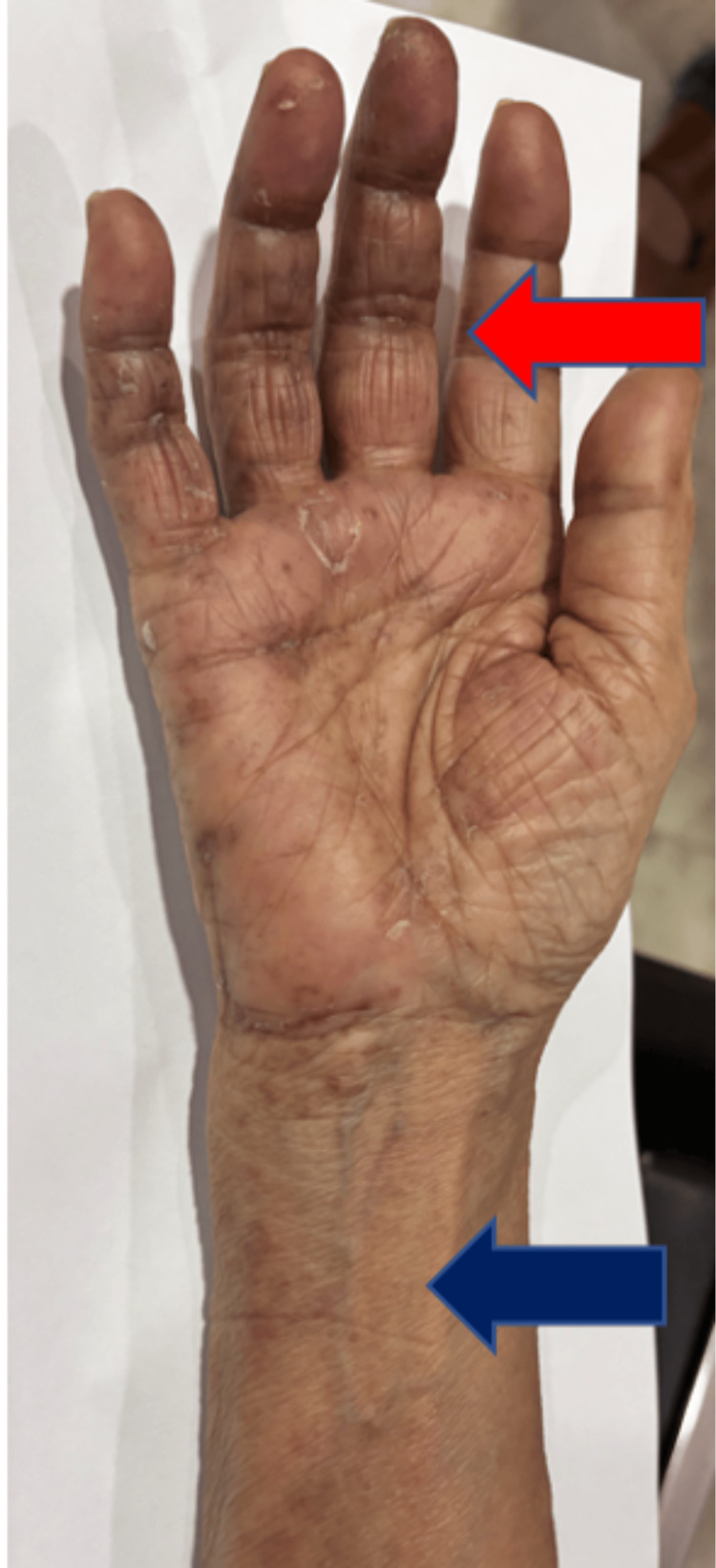
Healed hyperpigmented lesions and swelling over the right hand and forearm (C7/8 Dermatomes) Healed herpes rash with hyperpigmented lesions and swelling over the right hand (red arrow) and along the forearm (blue arrow)

**Figure 2:**
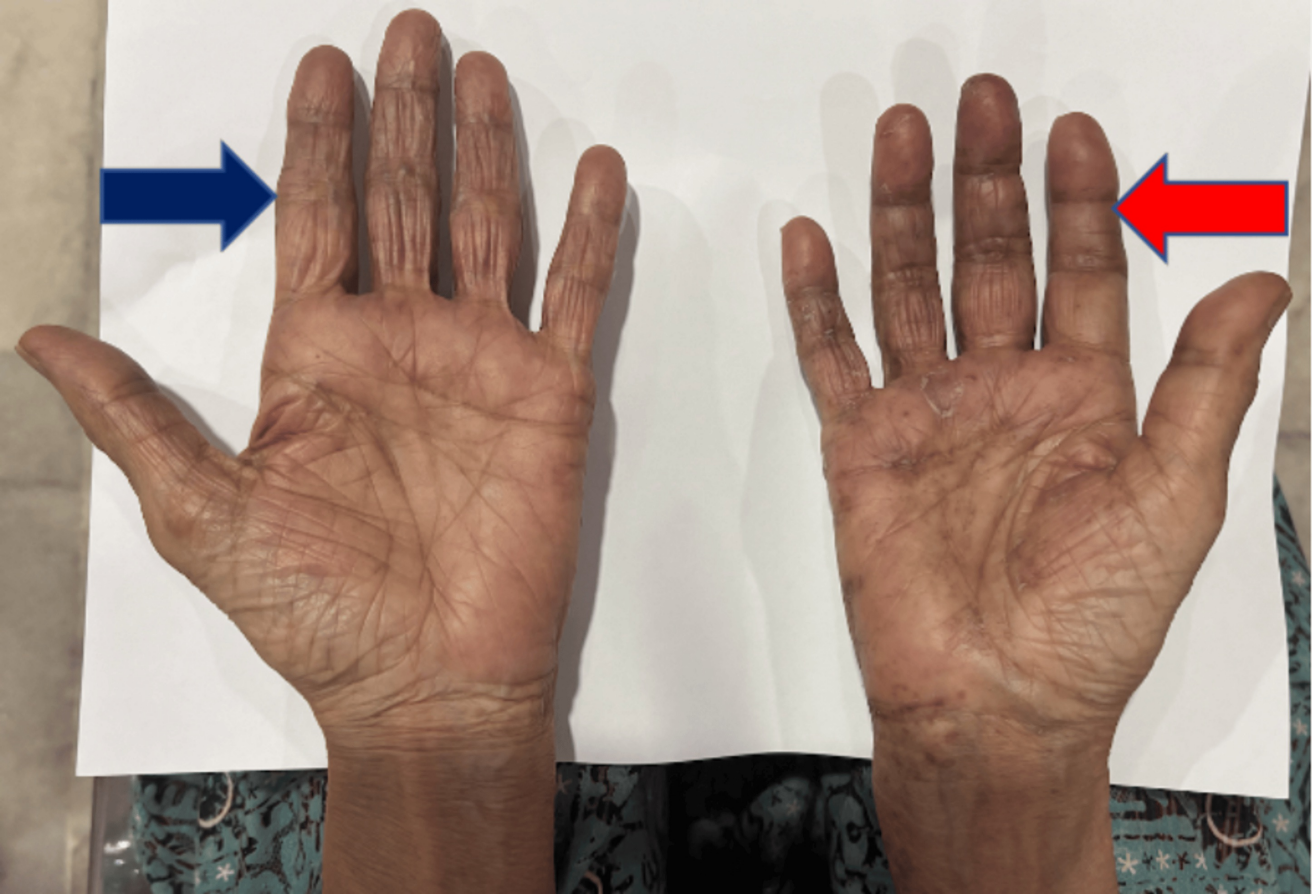
Comparison of unaffected and affected hands (C7/8 Dermatome) Comparison of unaffected (blue arrow) and affected hands (red arrow) demonstrating oedema and colour asymmetry.

Based on the Budapest diagnostic criteria and the clinical findings of pain, reduced range of motion, oedema, hypersensitivity, discolouration, and allodynia in the C7 and C8 dermatomes, we diagnosed her with CRPS (Figures 1, 2).

The patient was started on gabapentin, initiated at 300 mg once daily and gradually increased to 300 mg thrice daily (900 mg/day) over one week. She was also prescribed amitriptyline 10 mg at bedtime, along with paracetamol for pain relief. Physiotherapy was also initiated, including graded mobilisation, and range-of-motion exercises. On follow-up, the patient reported improvement in pain (VAS score 4/10).

## Discussion

CRPS is characterised by severe, disproportionate regional pain accompanied by sensory, vasomotor, sudomotor, and motor disturbances. It is typically classified into Types I and II. In Type I, there is no confirmed nerve injury, and Type II nerve lesion is usually present [1]. This usually follows trauma or surgery; however, non-traumatic triggers such as infections can also cause [2]. Pain intensity is usually assessed by the Visual Analog Scale (VAS) for the evaluation of both acute and chronic pain, as it is easy for the patient to describe the pain. The scale consists of a 10-cm line ranging from no pain (score 0) at one end and the worst pain possible (score 10) at the other, on which the patient indicates the perceived severity of pain [3].

Herpes zoster is a painful dermatomal vesicular eruption that is caused by the reactivation of latent VZV in the dorsal root ganglia of the spinal cord [4]. The most common complication, such as PHN, is characterised by persistent neuropathic pain following resolution of the rash, particularly in elderly or immunocompromised individuals [4]. Pathophysiology includes peripheral nerve damage due to varicella, and sensitisation can result from VZV-induced inflammation. This causes an increased expression of inflammatory mediators such as substance P, cytokines, and calcitonin gene-related peptide (CGRP). Through maladaptive plasticity in the spinal cord and supraspinal centres, central sensitisation increases pain even more due to abnormal nociceptive signalling. CRPS causes the dysregulation of the sympathetic nervous system, which, in turn, causes vasomotor abnormalities [5]. In CRPS, the affected patients have been shown to have elevated levels of pro-inflammatory cytokines like interleukin-6 (IL-6) and tumour necrosis factor-alpha. This is triggered by herpes zoster, which leads to CRPS [6]. CRPS can be clinically diagnosed by the Budapest criteria, which include signs and symptoms of sensory, vasomotor, sudomotor, and motor features [7].

A multidisciplinary approach is usually required for the treatment, which includes pharmacological therapies, physiotherapy, and interventional modalities [8]. Chemical or surgical sympathectomy is usually the treatment of refractory CRPS via ablation by radiofrequency waves, ultrasound-guided phenol injections of the sympathetic chain and the stellate ganglion [9]. If this is not achieved with either of the above, then open surgery is required. spinal cord stimulation, transcutaneous electrical nerve stimulation, the use of N-methyl-D-aspartate (NMDA) receptor antagonists, intravenous immunoglobulin, plasmapheresis, and antioxidants, particularly Vitamin C, are other modalities of treatment included in the literature [8,9]. Early antiviral medication has been demonstrated to reduce the severity of pain and viral replication in the context of herpes zoster [9,10]. In summary, CRPS is an uncommon but significant herpes zoster sequelae that is usually underdiagnosed. This case report emphasises the value of early detection and interdisciplinary treatment for the condition to reduce morbidity.

## Conclusions

CRPS is a rare sequelae of herpes zoster. There is an overlap between CRPS and PHN and hence very few cases are recognised in the community. This case highlights the significance of CRPS diagnosis after a zoster rash has cleared up and patient has persistent pain along with autonomic, sensory, and motor abnormalities. In order to prevent persistent disability and improve functional outcomes, early diagnosis is required. The Budapest criteria can be used in the diagnosis. Multidisciplinary treatment approach is essential in CRPS. To better understand the underlying mechanisms and improve therapeutic approaches, more research is needed.

## References

[1] Abd-Elsayed A, Stark CW, Topoluk N (2024). A brief review of complex regional pain syndrome and current management. Ann Med.

[2] Neumeister MW, Romanelli MR (2020). Complex regional pain syndrome. Clin Plast Surg.

[3] Bodian CA, Freedman G, Hossain S, Eisenkraft JB, Beilin Y (2001). The visual analog scale for pain: clinical significance in postoperative patients. Anesthesiology.

[4] Le P, Rothberg M (2019). Herpes zoster infection. BMJ.

[5] Liu Q, Han J, Zhang X (2024). Peripheral and central pathogenesis of postherpetic neuralgia. Skin Res Technol.

[6] Yan X, He Y, Yue Y, Zhang C, Yang H, Zhao Ph (2025). Post-herpetic neuralgia: review of pathophysiology, mechanisms, and drug treatment (online ahead of print). Curr Neuropharmacol.

[7] Harden NR, Bruehl S, Perez RS (2010). Validation of proposed diagnostic criteria (the "Budapest Criteria") for complex regional pain syndrome. Pain.

[8] Smart KM, Ferraro MC, Wand BM, O'Connell NE (2022). Physiotherapy for pain and disability in adults with complex regional pain syndrome (CRPS) types I and II. Cochrane Database Syst Rev.

[9] Singh H, Rajarathinam M (2024). Stellate ganglion block beyond chronic pain: A literature review on its application in painful and non-painful conditions. J Anaesthesiol Clin Pharmacol.

[10] Sampathkumar P, Drage LA, Martin DP (2009). Herpes zoster (shingles) and postherpetic neuralgia. Mayo Clin Proc.

